# Pulsatile Bi-Directional Aerosol Flow Affects Aerosol Delivery to the Intranasal Olfactory Region: A Patient-Specific Computational Study

**DOI:** 10.3389/fphar.2021.746420

**Published:** 2021-11-23

**Authors:** Ali Farnoud, Hesam Tofighian, Ingo Baumann, Andrew R. Martin, Mohammad M. Rashidi, Micheal P. Menden, Otmar Schmid

**Affiliations:** ^1^ Institute of Computational Biology, Helmholtz Zentrum München, Munich, Germany; ^2^ Comprehensive Pneumology Center, Member of the German Center for Lung Research, Munich, Germany; ^3^ Institute of Lung Biology and Disease, Helmholtz Zentrum München, Munich, Germany; ^4^ Mechanical Engineering Department, Amirkabir University of Technology, Tehran, Iran; ^5^ Department of Otorhinolaryngology, Head and Neck Surgery, Medical Center of Heidelberg University, Heidelberg, Germany; ^6^ Department of Mechanical Engineering, Faculty of Engineering, University of Alberta, Edmonton, AB, Canada; ^7^ Institute of Fundamental and Frontier Sciences, University of Electronics and Technology of China, Chengdu, China; ^8^ Department of Biology, Ludwig-Maximilians University Munich, Munich, Germany; ^9^ German Center for Diabetes Research (DZD e.V.), Munich, Germany

**Keywords:** nose to brain drug delivery, olfactory region, bi-directional aerosol delivery, pulsatile drug delivery, computational fluid dynamics, large eddy simulations

## Abstract

The nasal olfactory region is a potential route for non-invasive delivery of drugs directly from the nasal epithelium to the brain, bypassing the often impermeable blood-brain barrier. However, efficient aerosol delivery to the olfactory region is challenging due to its location in the nose. Here we explore aerosol delivery with bi-directional pulsatile flow conditions for targeted drug delivery to the olfactory region using a computational fluid dynamics (CFD) model on the patient-specific nasal geometry. Aerosols with aerodynamic diameter of 1 µm, which is large enough for delivery of large enough drug doses and yet potentially small enough for non-inertial aerosol deposition due to, e.g., particle diffusion and flow oscillations, is inhaled for 1.98 s through one nostril and exhaled through the other one. The bi-directional aerosol delivery with steady flow rate of 4 L/min results in deposition efficiencies (DEs) of 50.9 and 0.48% in the nasal cavity and olfactory region, respectively. Pulsatile flow with average flow rate of 4 L/min (frequency: 45 Hz) reduces these values to 34.4 and 0.12%, respectively, and it mitigates the non-uniformity of right-left deposition in both the cavity (from 1.77- to 1.33-fold) and the olfactory region (from 624- to 53.2-fold). The average drug dose deposited in the nasal cavity and the olfactory epithelium region is very similar in the right nasal cavity independent of pulsation conditions (inhalation side). In contrast, the local aerosol dose in the olfactory region of the left side is at least 100-fold lower than that in the nasal cavity independent of pulsation condition. Hence, while pulsatile flow reduces the right-left (inhalation-exhalation) imbalance, it is not able to overcome it. However, the inhalation side (even with pulsation) allows for relatively high olfactory epithelium drug doses per area reaching the same level as in the total nasal cavity. Due to the relatively low drug deposition in olfactory region on the exhalation side, this allows either very efficient targeting of the inhalation side, or uniform drug delivery by performing bidirectional flow first from the one and then from the other side of the nose.

## 1 Introduction

The nasal olfactory region is a potential non-invasive path for drug delivery to the brain for neurological disorders which by-passes the blood-brain barrier (BBB) (Frey et al., 1997; Mistry et al., 2009). Conventional nasal sprays provide large particles (>50 µm) which mainly deposit in the nasal valve and vestibule regions due to inertial impaction (Inthavong et al., 2011). Only a small fraction of spray aerosol passing through the anterior parts of the nasal cavity reaches the posterior region, where it either deposits in the lower parts of the nasal cavity or the turbinate regions (Cheng et al., 2001; Djupesland and Skretting, 2012), or it exits the nasal cavity into the nasopharyngeal region. Since the olfactory region is located in the upper posterior region, particles from conventional nasal sprays do not reach the olfactory region efficiently (Djupesland, 2013; Chen et al., 2020). This raises interest in new methods for targeting the olfactory.

It has been shown that nanoparticles are potentially more effective for aerosolized drug delivery to the nasal epithelium of the olfactory region than micron-sized particles. Several studies investigated the nose to brain delivery by transporting the drug-laden chitosan nanoparticles into the olfactory region (Alam et al., 2012; Chen et al., 2012; Fazil et al., 2012). Nanoparticle (<100 nm diameter, *d*_p_) delivery is considered potentially useful, since their diffusivity enhances aerosol transport into non- or poorly ventilated regions such as the olfactory epithelium region. In spite of this advantage, there are also severe limitations for nano-sized therapeutic aerosol. The main limitation arises from the cubic dependence of aerosol volume on particle diameter, which makes it difficult to deliver sufficiently high drug doses for therapeutic efficacy in patients with nanosized-sized aerosol. This becomes evident when considering that typically used 3 µm therapeutic aerosol has a 2.7 × 10^4^ fold higher drug (mass) dose than the same number concentration of 100 nm aerosol. Compensation of this drug loading deficiency would require a 2.7 × 10^4^ fold increase in 100 nm aerosol concentration (as compared to 3 µm aerosol). However, the resulting extremely high aerosol number concentrations (>10^10^ particle per cm^3^) would induce aerosol coagulation and thus an increase in aerosol size mitigating the desired diffusion-driven enhanced deposition efficiency in the olfactory region (Hinds, 1999).

Among the microparticles, the very fine particles (close to *d*_p_ = 1 µm) are preferred for olfactory aerosol delivery due to their relatively low relaxation time and high diffusivity which means that these particles follow the streamlines of the air flow (low impaction) and thus reach the olfactory region more than larger particles, where they experience enhanced deposition due to diffusion (Shi et al., 2006; Shi et al., 2007; Si et al., 2013). To overcome these limitations we investigate the potential of clinically already established pulsatile aerosol, in which the inhaled aerosol flow is superimposed with pressure oscillations resulting in enhanced ventilation and subsequently aerosol delivery to non- or poorly ventilated regions of the nose such as maxillary sinuses (Möller et al., 2014). Upon induction of pulsatile pressure in this cavity a standing pressure wave is formed, which results in cyclic flow into cavity at the resonance frequency of the cavity. Möller et al. (2014) have shown that while nasal sprays do not reach the non-ventilated paranasal sinuses, pulsatile aerosol devices such as the PARI SINUS nebulizer (PARI GmbH, Starnberg, Germany; mass median aerodynamic diameter, MMAD = 3 µm; pulsation frequency 45 Hz), or the DTF Aerodrug (Tours, France; MMAD = 3 µm; 100 Hz) deliver approximately 5% of the nebulized dose to the paranasal sinuses. Since the olfactory region is also a poorly ventilated region in the upper nasal cavity, it is conceivable that aerosol delivery with pulsatile flow may enhance aerosol transport onto the epithelium of the olfactory region.

**FIGURE 1:**
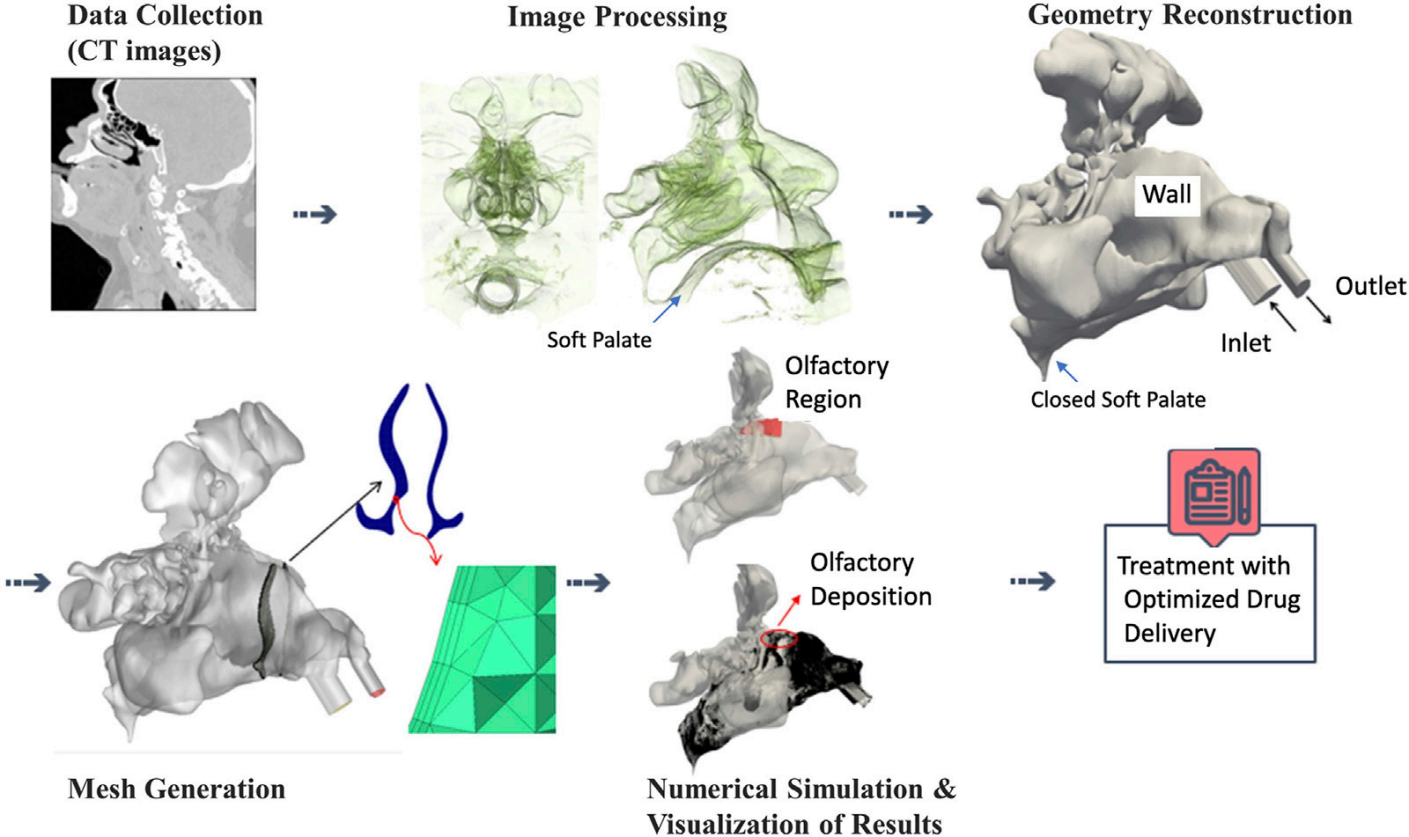
The workflow for modeling of patient-specific olfactory drug delivery starting from acquisition of CT images over geometry reconstruction and mesh generation to numerical simulation and visualization of particle-laden air flow (Farnoud et al., 2020a). For bi-directional aerosol flow, the soft palate needs to be closed, which is illustrated in top panels.

Bi-directional aerosol delivery methods are recently used to improve the deposition efficiency in the nasal cavity (Djupesland and Skretting, 2012; Xi et al., 2017). In contrast to normal (bilateral) inhalation during both nostrils, in bi-directional air flow technique (aerosol-laden) air flow is pushed with a pump into one nostril, flows to the end of the nasal cavity (nasopharynx), turns around into the other nasal cavity, and finally exits through the other nostril. This requires closing the soft palate (Figure 1), which separates the nasal from the oropharyngeal cavity. This can be accomplished by pushing the back part of the tongue towards the upper part of the oropharynx, while keeping the mouth open. As bi-directional flow constrains aerosol flow to the nose particle deposition in extranasal regions is prevented and particle deposition efficiency (DE) in the main nasal cavity is enhanced (Xi et al., 2018). During this process a certain fraction of the aerosol particles will deposit on the nasal wall—the rest is exiting the nasal cavity (Farnoud et al., 2020a). Bidirectional flow can also be established using devices such as the OptiNose (OptiNose AS; Oslo, Norway), which directs exhaled air from the mouth to the nostrils via a mouthpiece connected to a nasal interface (Djupesland and Skretting, 2012). An advantage of OptiNose as compared to inhalation-based nasal delivery systems (e.g., the PARI SINUS) is that during exhalation the soft palate is automatically closed. Thus, the patient does not have to learn how to close the soft palate, the OptiNose automatically enables bi-directional aerosol flow confined to the nose (Djupesland et al., 2006; Djupesland and Skretting, 2012; Farnoud et al., 2020a).

Bi-directional nasal aerosol deposition has not been studied extensively, but some experimental and numerical studies are available (Kleven et al., 2005; Xi et al., 2018; Hosseini and Golshahi, 2019). Xi et al. showed both experimentally and numerically that bi-directional nasal drug delivery enhances the deposition of particles in the upper portion of the nasal cavity (Xi et al., 2018), especially in the olfactory region (Xi et al., 2017). Due to the relatively poor ventilation of that part of the nasal cavity which is in close proximity to the epithelium of the olfactory region, the diffusive nature of nanoparticles (<100 nm diameter) may provide a means for enhanced drug delivery to the olfactory epithelium. While nanoparticles have also been explored with bi-directional administration for targeting the olfactory region (Tian et al., 2017), use of nanoparticles does not allow large aerosol mass doses to be inhaled. Hence, in the present study we explore the size range between traditional aerosol for pulmonary drug delivery (3–5 µm diameter) and nanoparticles, namely 1 µm particles, for its capacity of enhanced drug delivery to the olfactory region.

The present computational fluid dynamics study investigates the regional deposition pattern and DE of 1 µm particles in the entire nasal airway, and specifically in the olfactory region, during bi-directional delivery into realistic nasal geometry. Two-way coupling between particles and air flow are considered and flow partitioning in the olfactory region and the effect of aerosol pulsation on olfactory drug delivery are analyzed. Since the local drug dose per area determines the therapeutic efficacy of a drug, in this study regional dose in the nasal cavity and specifically olfactory region is investigated as mass per area. Moreover, dose and DE in right and left cavity and olfactory regions are compared for pulsatile and non-pulsatile inlet flow conditions.

## 2 Methodology

A three-dimensional geometry of the nasal cavity and paranasal sinuses was reconstructed from CT images of a male, 82-year-old patient, as previously studied by Farnoud et al. (2017), Farnoud et al. (2020a), and Farnoud et al. (2020b). The CT images were segmented using the open source software package 3DSlicer. The paranasal sinuses are healthy and the diameters of right and left ostia are 4.58 and 5.45 mm. The software package of ICEM-CFD (Ansys) was used for the volume grid generation. Here approximately 20 million unstructured tetrahedral cells with 3 prism layers were generated, which is required to capture correct large eddy simulations (LES) as well as particle deposition on the wall. The patient was asked to keep his mouth open by holding a pipe between his teeth during CT imaging; therefore, the three-dimensional geometry represents the realistic closed soft palate condition which is ideal for bi-directional drug delivery in nasal cavity. Following recommendations from the literature, and the manual of the devices such as PARI SINUS nebulizer, two clockwise 45° nosepieces were connected to the nostrils (Farnoud et al., 2020a). Figure 1 illustrates the workflow for CT-based simulation and visualization of particle-laden airflow inside nasal cavity.

### 2.1 Governing Equations

#### 2.1.1 Continuous Phase Equations

In the present model, the three-dimensional, incompressible unsteady Navier-Stokes equations in isothermal condition are discretized and numerically solved. A LES turbulence model is implemented for simulation of the fluid flow which may be laminar, transitional, or turbulent. Furthermore, a two-phase flow model was used for considering the interaction between airflow and particles.

The three-dimensional unsteady flow field inside the nasal airway is governed by the isothermal filtered incompressible Navier-Stokes equations as:

$$\frac{\partial {\tilde{u}}_{i}}{\partial x_{i}}=0,$$ (1)

$$\frac{\partial {\tilde{u}}_{i}}{\partial t}+\frac{\partial \left({\tilde{u}}_{i}{\tilde{u}}_{j}\right)}{\partial x_{j}}=-\frac{1}{\rho }\frac{\partial \tilde{p}}{\partial x_{i}}+\frac{\partial }{\partial x_{j}}\left[2\left(\nu +\nu_{\text{SGS}}\right){\tilde{S}}_{ij}\right]+\frac{1}{\rho }{\tilde{S}}_{u_{i}},$$ (2)

where implicit grid filter operator $\tilde{\;}$ has the local characteristic spatial length of $\tilde{\text{Δ}}={\left(V_{cell}\right)}^{1/3}$ and $V_{cell}$ is the volume of the computational cell. $\tilde{u}$ is the filtered velocity, $\tilde{p}$ is the filtered pressure, t is time, x is the spatial coordinate, and ρ is the gas density. ν and $\nu_{\text{SGS}}$ are laminar and Sub-Grid Scale (SGS) kinematic turbulent viscosities, respectively, and $\tilde{S}$ is the filtered strain-rate tensor. ${\tilde{S}}_{u_{i}}$ is the source term in the momentum equation and is generated due to the interaction between gas phase and particles.

The LES turbulence model adds $\nu_{\text{SGS}}$ in Eqn. to reproduce filtered eddies motions effect as a diffusion process. Moreover, $\nu_{\text{SGS}}$ is calculated via a transfer equation for SGS kinetic energy ( $k_{\text{SGS}}$ ) as follows:

$$\nu_{\text{sgs}}=C_{k}\tilde{\Delta }k_{\text{sgs}}^{1/2},$$ (3)

$$\frac{\partial k_{\text{SGS}}}{\partial t}+{\tilde{u}}_{j}\frac{\partial k_{\text{SGS}}}{\partial x_{j}}=\nu_{\text{SGS}}\left(2{\tilde{S}}_{ij}{\tilde{S}}_{ij}\right)-C_{\varepsilon }k_{\text{SGS}}^{3/2}/\tilde{\Delta }+\frac{\partial }{\partial x_{j}}\left(\nu_{\text{SGS}}\frac{\partial k_{\text{SGS}}}{\partial x_{j}}\right).$$ (4)

The values of $C_{k}$ and $C_{\varepsilon }$ are dynamically calculated with the help of a second filter with length of $\hat{\text{Δ}}=2\tilde{\text{Δ}}$ . The sub-grid dynamic stress tensor yields

$$T_{ij}=\hat{\tilde{u_{i}u_{j}}}-{\hat{\tilde{u}}}_{i}{\hat{\tilde{u}}}_{j}.$$ (5)

The difference between the turbulent sub-grid dynamic stress tensor and turbulent SGS tensor is described by Germano identity:

$$L_{ij}=T_{ij}-{\tilde{\tau }}_{ij}=\hat{{\tilde{u}}_{i}{\tilde{u}}_{j}}-{\hat{\tilde{u}}}_{i}{\hat{\tilde{u}}}_{j},$$ (6)

where $L_{ij}$ is obtained from direct solution and $C_{k}$ and $C_{\varepsilon }$ are calculated based on the dynamic method provided by Kim and Menon:

$$C_{k}=\frac{\langle L_{ij}M_{ij}^{k}\rangle }{\langle M_{ij}^{k}M_{ij}^{k}\rangle }\;,$$ (7)

$$C_{\varepsilon }=\frac{\langle \xi m\rangle }{\langle mm\rangle },$$ (8)

$$M_{ij}^{k}=\hat{\tilde{\text{Δ}}{\tilde{k}}^{1/2}\left|\tilde{S}\right|}-2\hat{\text{Δ}}{\hat{\tilde{k}}}^{1/2}\left|\hat{\tilde{S}}\right|,$$ (9)

where $\xi =L_{ii}$ , $m={\hat{\tilde{k}}}^{3/2}/\hat{\text{Δ}}-\hat{{\tilde{k}}^{3/2}/\tilde{\text{Δ}}}$ and 〈⋅〉 shows the volume average of test filter.

In addition to the filtered Navier-Stokes equations, an equation for the conservation of the passive scalar which is entering from the inlet as follows:

$$\frac{\partial \tilde{c}}{\partial t}+\frac{\partial \left({\tilde{u}}_{j}\tilde{c}\right)}{\partial x_{j}}=\left(\frac{\nu }{\text{Sc}}+\frac{\nu_{SGS}}{{\text{Sc}}_{\text{SGS}}}\right)\frac{\partial^{2}\tilde{c}}{\partial x_{j}^{2}},$$ (10)

where *c* is the concentration of the passive scalar that enters the computational domain through the right nosepiece. Sc and ${\text{Sc}}_{sgs}$ are Schmidt number and sub-grid turbulent Schmidt numbers respectively.

#### 2.1.2 Dispersed Phase Equations

The Lagrangian solver at time *t* calculates a total number of N computational particles (parcels) as $\left\{x_{\text{p}}\left(t\right),\;u_{\text{p}}\left(t\right),d_{\text{p}},\rho_{\text{p}},\omega_{\text{p}};\text{p}=1,\ldots ,N\left(t\right)\right\}$ where $x_{p}$ is the position vector and $u_{\text{p}}$ is the velocity vector and $d_{\text{p}}$ , $\rho_{\text{p}}$ , and $\omega_{\text{p}}$ are diameter, density, and the statistical weight of the *p*th particle. The following Lagrangian equations update the position and velocity of the particles in each time step by exerting drag and gravitational forces on the particles.

$$\frac{dx_{\text{p}}}{dt}=u_{\text{p}},$$ (11)

$$\frac{du_{\text{p}}}{dt}=\frac{u_{\text{s}}-u_{\text{p}}}{\tau_{\text{p}}}+g.$$ (12)

In these equations g is the gravitational acceleration and $u_{\text{s}}$ is the instantaneous gas velocity seen by the particle and $\tau_{p}$ is the particle relaxation time scale which is calculated as follows:

$$\tau_{\text{p}}=\frac{\tau_{\text{p},St}}{f_{\text{d}}},\;\;\;\;\;\;\;\tau_{\text{p},St}=\frac{\rho_{\text{p}}d_{\text{p}}^{2}}{18\mu },$$ (13)

$f_{\text{d}}$ is the drag coefficient that is calculated using Schiller and Neumann correlation:

$$f_{d}=\{\begin{array}{l} 1\;\;\;\;\;\;\;\;\;\;\;\;\;\;\;\;\;\;\;\;\;\;\;\;\;\;\;\;\;\;\;\;\;\;\;\;\;\;\;\;\;\;\;\;\;\;;{\text{Re}}_{\text{p}}\leq 1\\ \frac{24}{Re_{p}}\left(1+0.15\;{Re}_{p}^{0.678}\right)\;\;\;\;\;\;\;;1<{\text{Re}}_{\text{p}}\leq 1000\\ 0.44\;\;\;\;\;\;\;\;\;\;\;\;\;\;\;\;\;\;\;\;\;\;\;\;\;\;\;\;\;\;\;\;\;\;\;\;\;\;\;\;\;;{\text{Re}}_{\text{p}}>1000 \end{array}$$ (14)

where $Re_{\text{p}}$ is the particle Reynolds number and is defined as $Re_{\text{p}}=\left|u_{\text{s}}-u_{\text{p}}\right|d_{\text{p}}/\nu$ .

Although the true value of the instantaneous gas velocity seen by the particle can only be obtained by direct numerical solution (DNS), the effect of the modeled fluctuations will be negligible due to the very fine grid used in the present work, and therefore, the value of $u_{\text{s}}$ used in Eq. 11 is predicted by the interpolation of the averaged velocity of $\tilde{u}$ in the location of each parcel.

In each time step the source term $S_{u_{i}}$ in Eq. 2 is calculated in the l th cell as follows:

$${\left[S_{u_{i}}\right]}^{\left[l\right]}=\frac{1}{\text{Δ}t\forall_{\text{C}}^{\left[l\right]}}\overset{N}{\underset{\text{p}=1}{\sum }}\left(G_{l}\left(x_{\text{p}}\right)\omega_{\text{p}}F_{\text{D},i}^{\text{p}}\right),$$ (15)

Δt in Eq. 15 is the time step, $\;\forall_{\text{C}}^{\left[l\right]}$ is the volume of the volume grid cell, and $G_{l}\left(x\right)$ is the linear weighted interpolation kernel function, and $F_{\text{D},i}^{\text{p}}=m_{\text{p}}\left(u_{\text{s},i}-u_{\text{p},i}\right)/\tau_{\text{p}}$ is the drag exerted on *p*th parcel; the summation in Eq. 15 is applied on all parcels.

### 2.2 Numerical Methods

The open-source software package of OpenFOAM (www.openfoam.org) is used to numerically solve the particle-laden flow equations inside the nasal airway. The Pressure-Implicit with Splitting of Operators (PISO) method combined with Semi-Implicit Method for Pressure-Linked Equations (SIMPLE) algorithm (pimpleFoam solver) is used for the solution of the Eulerian phase and the Lagrangian Intermediate library is combined with the pimpleFoam solver to enable the solution of the secondary phase in the gas phase (Patankar and Spalding, 1972; Patankar, 1980; Syrakos et al., 2017). The pimpleFoam solver is suitable for LES simulations with the local dynamic k-equation sub-grid scales (SGS) model. The local dynamic k-equation SGS model is used since it covers the laminar-transitional-turbulent region so that it is very suitable for the simulation of the present work which is predominantly laminar with possible transitional and turbulent flow regimes. It is important to note that in the local dynamic k-equation SGS model, $C_{k}$ and $C_{\varepsilon }$ is dynamically chosen depending on the time and space.

The diffusion terms are discretized with the least-squares scheme, which is second-order accurate on general unstructured meshes (Martínez et al., 2015). Since upwind-dominated schemes could lead to numerical dissipations in orders of SGS viscosity dissipation and consequently decrease the precision of the numerical simulations (Sweby, 1984), the convection term in the momentum equation requires a proper discretization scheme. In the present work, “Gauss filteredLinear” scheme, which is a low-dissipation second-order central differencing method, is implemented for LES. The convection term of other (scalar) quantities is discretized by the second-order “Gauss limitedLinear” scheme based on Total Variation Diminishing (TVD) (Moukalled et al., 2016). The time derivatives are discretized by the Second-Order Upwind Euler (SOUE) scheme (Wang et al., 2009).

Based on Figure 1, the nasal cavity is divided into three main parts: the wall, inlet, and outlet. The nosepieces are extended to represent the fully developed condition (Shi et al., 2007). The sinusoidal and steady air flow profiles are implemented for the velocity at the inlet by a Dirichlet boundary conditions. The air velocity for both inlet conditions represents an average flow rate of 4 L/min. The velocity profile of the gas phase is described as

$$U=U_{0}+U_{0}sin\left(2\pi \omega t\right),$$ (16)

where ω is 45 Hz. OpenFOAM utilizes a phase by face-to-face particle tracking procedure to simulate the motion of the particles (Macpherson et al., 2009). The analytical method is used to integrate particle Lagrangian equations (Minier et al., 2003). Furthermore, the properties of water are assigned to the particles since most of the drugs are aqueous solutions and as a result their densities are close to water density (Edwards et al., 1997). Utilizing the so-called parcel method, 4 million computational particles (parcels) with a total injection mass of 2.5 mg are uniformly and randomly entering through the right nosepiece for a duration of 0.5 s to represent realistic conditions as encountered for nebulizers such as PARI SINUS in contrast to the majority of the research in the literature which only considers a very short injection duration in range of a single time step. Moreover, two-way coupling between particles and gas phase is considered due to the high volume fraction of the particles of 7.5 E-5, which lies above the threshold of 1 E-6 (Ferrante and Elghobashi, 2007; Crowe et al., 2011). Since two-way coupling between air momentum and particles is considered, a particle-related source term is added to the gas phase momentum equation. In order to avoid statistical error, a large number of particles are required to be injected (Tofighian et al., 2019). In the literature typically short injections are studied and one-way coupling is implemented for simulation of drug delivery with different devices (Ahookhosh et al., 2021; Taheri et al., 2021); however, for the simulation of the aerosol delivery with nebulizers which have continuous cloud injection, one-way coupling between both phases does not require a particle-related source term in the momentum equation. The introduction of the source term requires larger statistical power, which requires an injection of a few million particles as compared to the previously used 10,000–50,000 particles (Dastan et al., 2014; Ghahramani et al., 2014; Keeler et al., 2016). Moreover, due to deposition of very few particles in the olfactory region, an injection with large number of particles is required to achieve independency of DE in olfactory region from number of injected particles and consequently to avoid statistical errors in prediction of olfactory epithelium deposition.

Here, 1 µm particles are considered with an average flow rate of 4 L/min. Moreover, pulsatile aerosol delivery is compared with non-pulsatile (steady) inlet airflow carrying particles with a diameter of 1 µm. As mentioned above, this particle size is used due to its relatively low relaxation time and high diffusivity which means that particles follow the streamlines (low impaction) and reach the olfactory region where they can deposit due to diffusion more than larger particles. This concept also rationalizes the choice of a relatively low flow rate of 4 L/min, since this reduces impaction in bends like vestibule and nasal valve and enhances the residence time and thus diffusive deposition of the particles in the olfactory region. The particles were distributed randomly and uniformly across the inlet plane and the velocities of the particles at the inlet are equal to the instantaneous velocity of the gas phase at the specific time and location of each particle. It is assumed that the particles stick to the wall once they touch it. The particles are followed until they hit the wall or reach the outlet and escape the computational domain. Although aerosol injection ceases after 0.5 s, the computational simulation is continued for 1.98 s to ensure that all particles have either deposited or exited the domain. The simulations are performed using 256 CPUs and the time steps were variably chosen based on the Courant number criteria to be lower than 1 which led to time steps in a range of 10^−5^ s to 10^−6^ s.

## 3 Results and Discussion

### 3.1 Model Validation for Regular (Bilateral) Inhalation

The validity of the present numerical solver was assessed in two steps. In our previous publications (Farnoud et al., 2017; Farnoud et al., 2020a; Farnoud et al., 2020b), the DE in 90° bends was compared to the experimental data of Pui et al. (1987) and other research in the literature (Breuer et al., 2006; Nicolaou and Zaki, 2016; Inthavong, 2019) to ensure that the solver is able to capture the DE of particles correctly. This benchmark is a popular approach to validate the capability of a numerical solver to predict particle deposition in curvature geometries.

**FIGURE 2:**
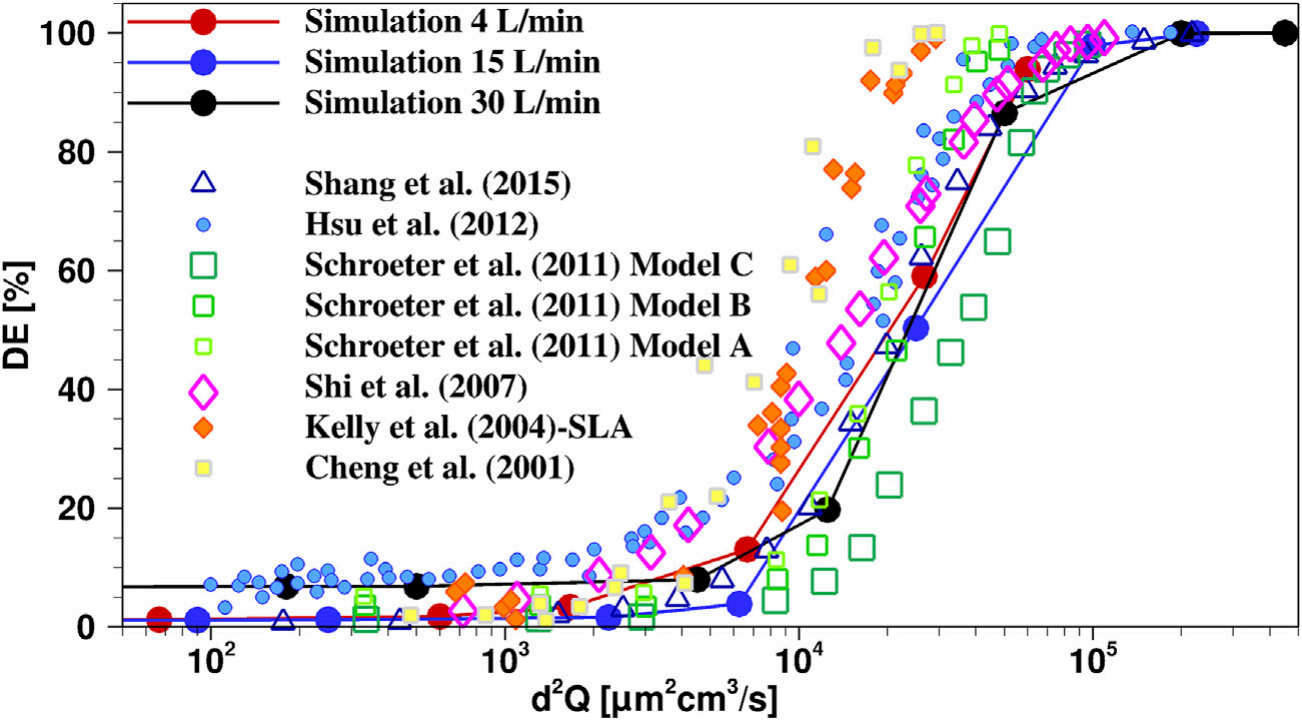
Comparison of present “simulation” results with experimental (Cheng et al., 2001; Kelly et al., 2004; Schroeter et al., 2011; Hsu and Chuang, 2012) and numerical (Shi et al., 2007; Shang et al., 2015) studies in literature from different realistic nasal geometries and similar boundary and initial conditions. The 1 µm case is represented by the lowest d^2^Q value for each of the flow rate curves.

In the next step, our model was compared to experimental and numerical data obtained for other nasal passages. Since the majority of the experimental and numerical studies on nasal aerosol delivery have focused on bilateral aerosol delivery, we validated our computational model with experimental DE data from a different nasal passage with bilateral drug delivery condition. Thus, for the current nasal passage geometry with bilateral air flow (drug delivery), open soft palate and delivery through both nostrils, DE is calculated as a function of the characteristic impaction parameter ( $\text{IP}=d^{2}Q$ ) and compared with previous nasal drug delivery studies in the literature (Cheng et al., 2001; Kelly et al., 2004; Shi et al., 2007; Hsu and Chuang, 2012). Moreover, in our previous study (Farnoud et al., 2020a) the pressure drop in nasal passage was compared to pressure drop in other patient-specific nasal airway models which showed a good agreement between the studies (Kelly et al., 2004; Schroeter et al., 2011). For mono-disperse particles with diameters of 1–30 µm (100,000 particles launched for each case) carried by three airflow rates of 4, 15, 30 L/min entering through both nostrils (regular inhalation), the calculated DEs agree well with experimental data and other computational studies in the literature that used different nasal airways and similar boundary and initial conditions (see Figure 2) (Cheng et al., 2001; Kelly et al., 2004; Shi et al., 2007; Hsu and Chuang, 2012). It is also important to note that for 1 µm at 4 L/min the DE is with 1.25% rather low indicating relatively low particle deposition due to impaction and diffusion. This is consistent with the well-known nasal incapability limit of ca. 10 µm, i.e., for normal breathing only particles smaller than ca. 10 µm can reach the lung (Hinds, 1999).

### 3.2 Mesh Quality Control

**FIGURE 3:**
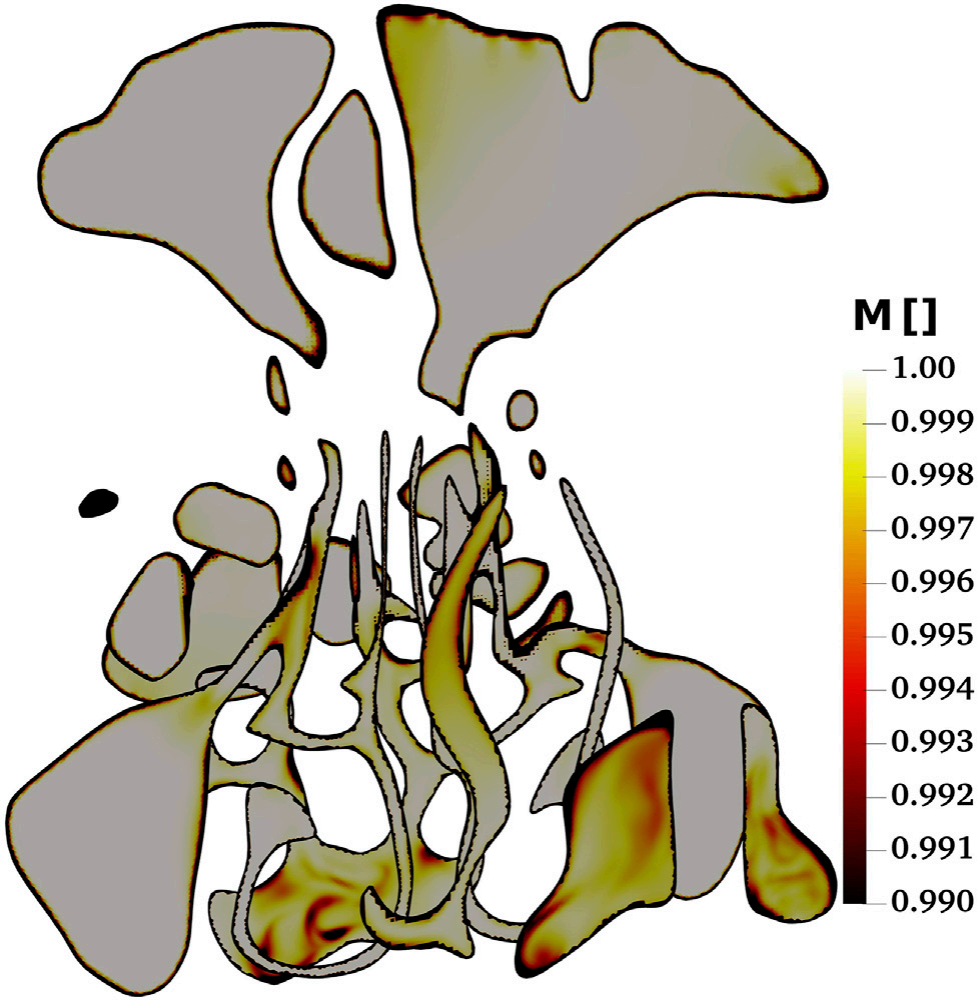
The contour plots of ratio of resolved turbulent kinetic energy (TKE) to total TKE as a control for the mesh quality of the present large eddy simulations (LES) study.

The result of large eddy simulation (LES) is highly dependent on the quality and resolution of the computational grid. To determine the resolution and quality of a LES simulation various criteria, typically referred to as “LES indices of Resolution Quality” (LES_IQk), have been introduced. One LES_IQk is defined as the ratio of resolved turbulent kinetic energy (TKE) to the total TKE, and can be formulated as:

$$M=k_{\text{res}}/\left(k_{\text{res}}+k_{\text{sgs}}\right)$$ (17)

where $k_{\text{res}}$ s is the resolved turbulent kinetic energy and $k_{\text{sgs}}$ is the sub-grid scale part of the turbulent kinetic energy. Celik et al. (2005) recommended a range of 0.77–0.85 for *M* to ensure that the grid resolution is adequate. Pope (2004) also considered grid resolution as sufficient if *M* is larger than 0.8. It is evident from Figure 3 that *M* is higher than 0.99 in the entire computational domain, indicating that the grid is finer than typically required. This hyperfine grid resolution is chosen since the fine-grained and complex geometry of the nasal cavity and relatively high flow rates require such a fine mesh.

### 3.3 Profiles of Bi-Directional, (Non-)Pulsatile Air Flow

In the present study, the effect of bi-directional pulsatile inlet airflow on the deposition pattern of the particles in the nasal airway, and specifically the olfactory epithelium region, is investigated. Since the inlet airflow profile directly affects dispersion and deposition of the particles, pulsating and non-pulsating airflow conditions are implemented at the inlet to assess the effect of the pulsation on the airflow pattern inside the nasal airway and specially the olfactory region.

**FIGURE 4:**
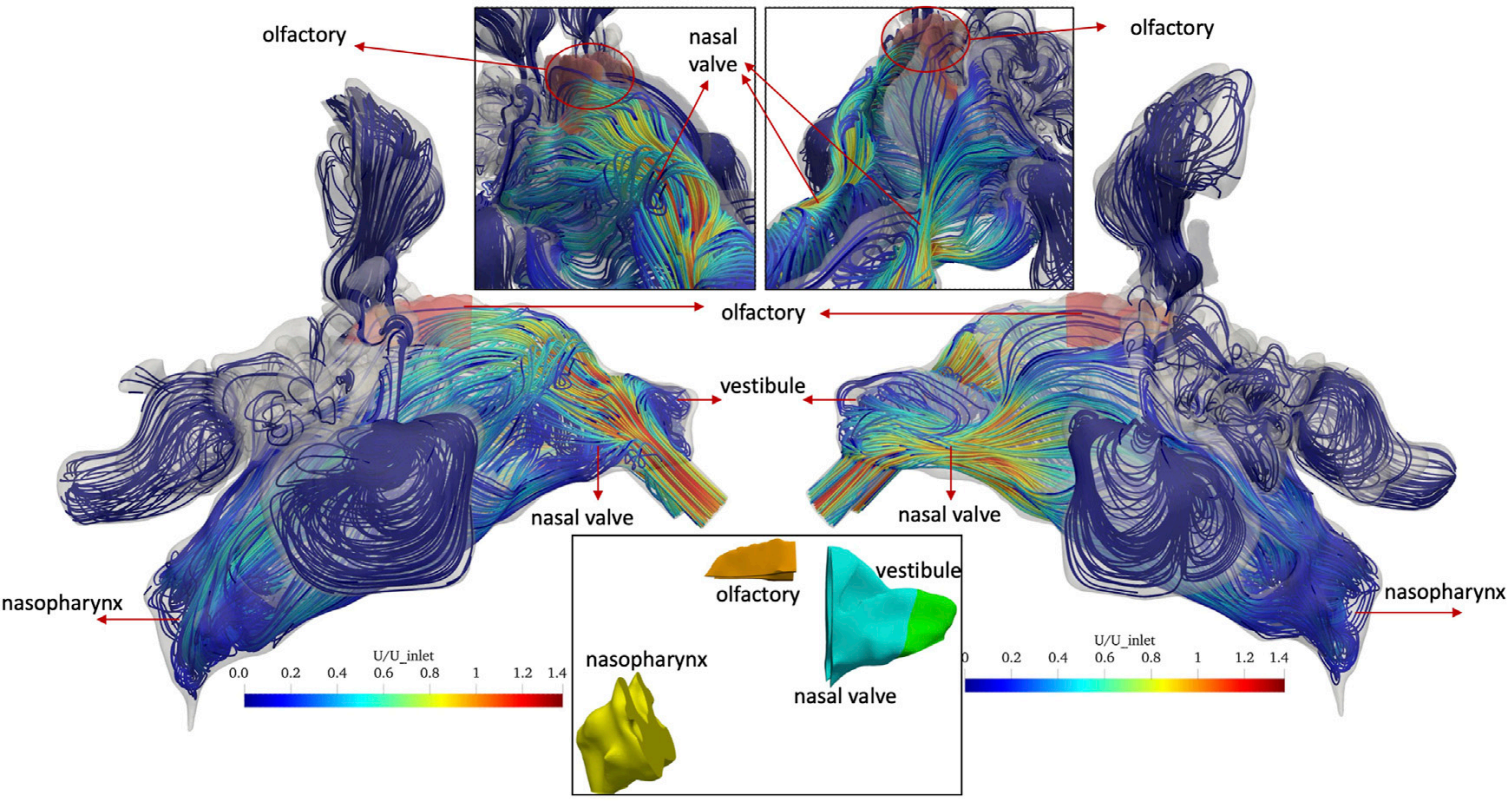
Streamlines of the normal(-ized) air velocity U in the nasal cavity for *non-pulsatile* (constant) inlet flow of 4 L/min at the end of inhalation period (*t* = 1.98 s). Right (inlet) and left (exit) views and close-up views of the olfactory region (shaded in red) are given in the left and right panel, respectively. In the bottom panel, different regions of the nasal anatomy are shown.

Figure 4 shows the streamlines of the air with non-pulsatile (constant) inlet flow rate of 4 L/min which are colored by normal velocity (normalized to average air speed at the inlet, i.e., right nostril). The simulations are performed until *t* = 1.98 s and Figure 4 represents the results at this time. Different regions of the nasal anatomy are shown in the bottom panel of Figure 4 to enable the further discussion based on the regions in the nasal cavity. At the right vestibule, a recirculation zone is observed and swirling flows occur as the air passes through the nasal valve. At the upper parts of the nasal valve (Figure 4. Top left panel) a swirling flow is observed which moves downwards to the inferior right meatus. The air flow from the nasal valve is divided into three meatuses and some streamlines move towards the top of the nasal cavity and pass through the olfactory region with relatively high velocity. At the nasopharynx the airflows from the three meatuses mix and make a 180° turn while entering the left cavity. At the left cavity, the flow moves from the nasopharynx towards the left nostril and uniform streamlines are observed in the entire main nasal airway except for the right vestibule where a recirculation zone is observed. In both right (entering) and left (exiting) vestibules recirculation zones are observed. The air velocity at the left vestibule increases since the upstream airflow encounters a sudden contraction in the nasal valve region.

**FIGURE 5:**
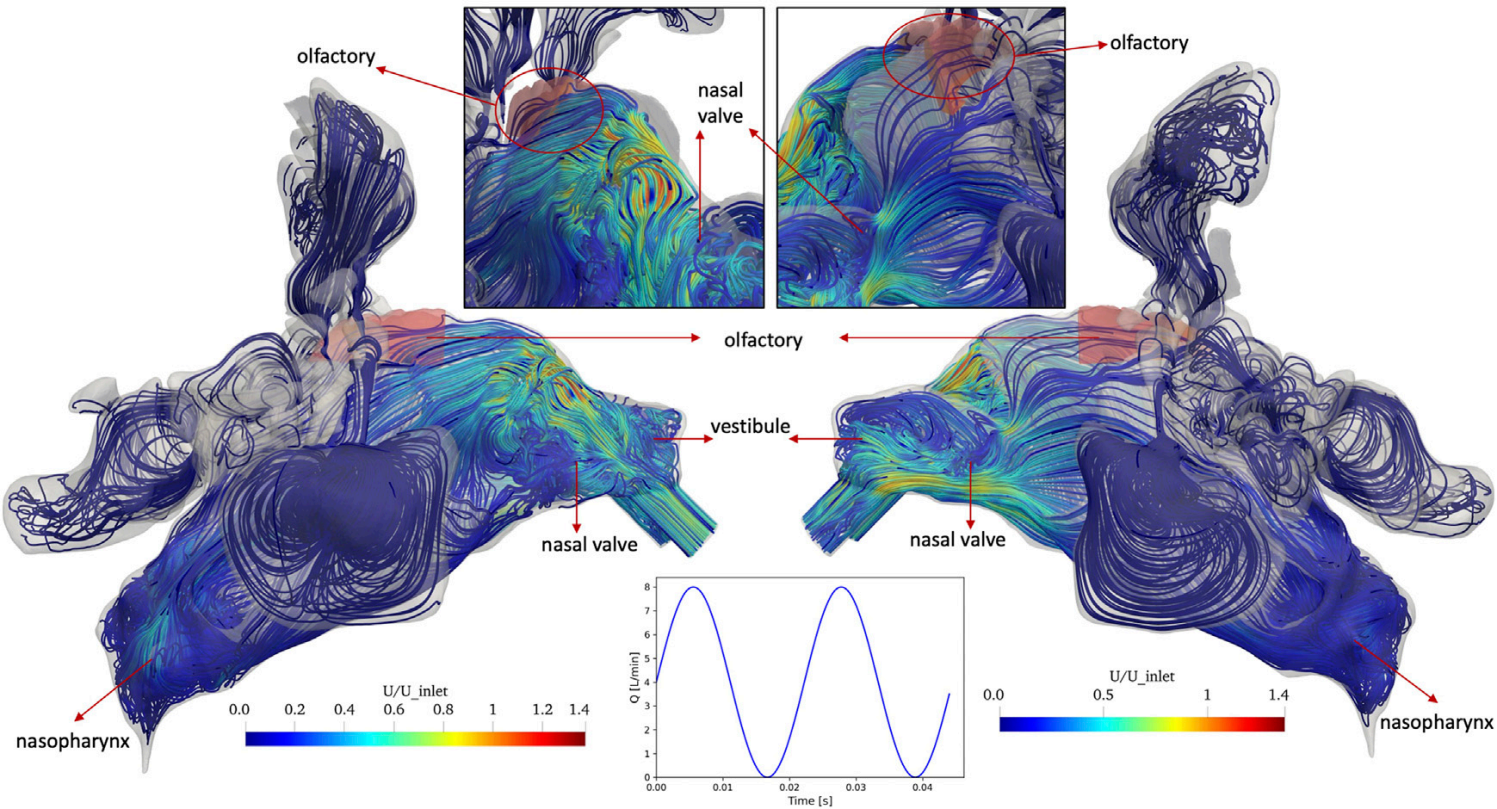
Streamlines of the normal(-ized) air velocity U in the nasal cavity for *pulsatile* inlet flow of 4 L/min with a frequency of 45 Hz (oscillation period T = 22.2 ms) at T/2 of the last modeled pulse (*t* = 1.98 s), where T/2 (middle of cycle of air flow pulsation) represents the condition where the inlet flow is equal to the average flow rate of the pulsatile flow profile. Again, the olfactory epithelium region is shaded in red.

Similarly, Figure 5 shows the streamlines of the normal velocity in the nasal cavity and olfactory region at T/2 of the last air flow pulse for the pulsatile inlet airflow with average flow rate of 4 L/min and frequency of 45 Hz (oscillation period: T = 22.2 ms). A complex airflow pattern including recirculation zones and swirling flows are observed in the anterior parts of both right and left main nasal airway due to periodical pulses in the airflow. For both inlet airflow conditions, the maximum velocity is observed in the anterior region and is close to 1.4 times the average inlet velocity. In contrast, streamlines with higher velocities pass through the right olfactory region when the non-pulsatile airflow is implemented at the inlet. The pulsatile airflow at the inlet avoids the impingement of the airflow to the top of the cavity after the nasal valve, which results in streamlines with much lower velocity passing the olfactory region.

Since the streamlines only show the flow pattern and not the amount of the air reaching to each region, the airflow fraction in the right and left olfactory regions are calculated. For this purpose, a new variable, the concentration of a passive scalar tracer (C), is introduced in the OpenFOAM code. C is initially 0 in the nasal cavity but changes to 1 when the drug-laden air flow reaches the specific computational cell. Analogous to a gaseous tracer, once all of the initially present air in the nasal cavity is replaced by the inhaled air, C = 1 throughout the nasal cavity. Hence, C reflects the fractional replacement of the initially present air with the inhaled air (drug-laden air fraction). The variable C is calculated in the whole domain including non-convectively ventilated paranasal sinuses and the olfactory region.

**FIGURE 6:**
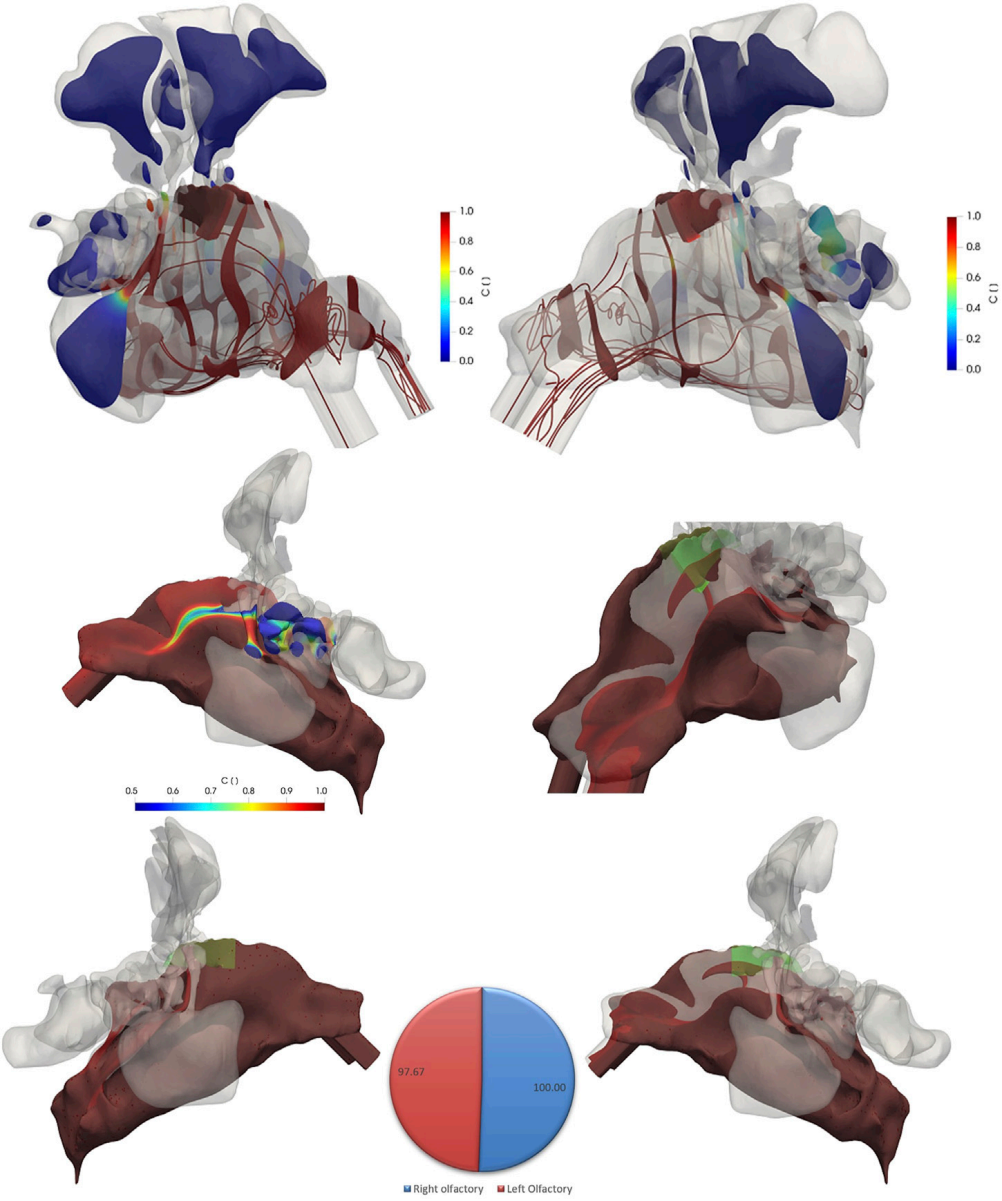
The contour plots of variable C (drug-laden air fraction) and flow streamlines (color coded for C) in different regions of the nasal cavity specifically in the olfactory region (green shaded) at *t* = 1.98 s (end of delivery phase) for the non-pulsatile inlet flow rate of 4 L/min (top figures). The figures at the bottom show the iso-volumes of variable C for values more than 0.99 from left and right views as well as close-up view of the left cavity. An iso-volume of variable C with values more than 0.5 is shown in the middle figure for a better visualization of values. The Pie chart shows the volume weighted value of C in the left and right olfactory regions. The green shaded regions in the bottom figures represent the olfactory region.

Figure 6 shows the contour plots of the variable C and the streamlines of the air flow (at the end of inhalation period t = 1.98 s) colored by variable C in different regions of the nasal cavity specifically in the olfactory region for *non-pulsatile* inlet airflow condition. It is noteworthy that C is larger than zero even in some regions of the non-ventilated paranasal sinuses (Figure 6, top), which is due to diffusional (not convective) transport of inhaled air into these regions. Moreover, Figure 6 (bottom) depicts the iso-volumes (regions that represent a specific value of a parameter) of variable C for values more than 0.99 which means 99% of these regions are filled with inhaled air indicating that these regions can be readily reached by the inhaled air either via convection or via diffusion. As even the lowest iso-volumes of C = 97.67% in the left nasal valve and vestibule are close to 100%, gas exchange in the entire nasal cavity (including olfactory epithelium region) is highly effective for a 1.98 s inhalation (bottom panel and pie chart in Figure 6). This implies that also olfactorially relevant gas molecules carried by the inhaled air will be able to efficiently reach the olfactory epithelium, which is a prerequisite for the sense of smell to work properly. For fine particles with low relaxation time and high diffusivity, the same statement is valid.

**FIGURE 7:**
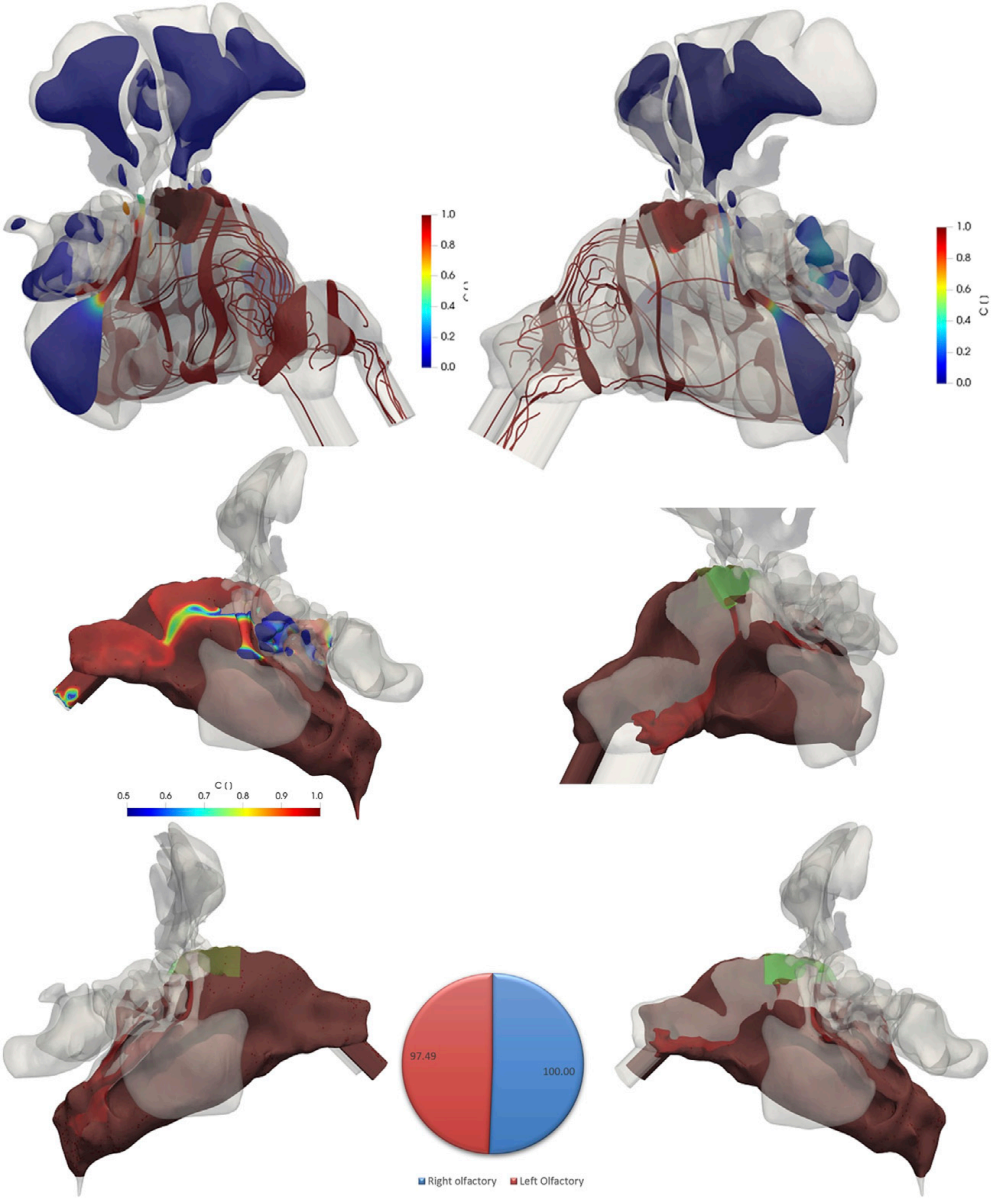
The contour plots of variable C (drug-laden air fraction) and flow streamlines (color coded for C) in different regions of the nasal cavity specifically in the olfactory region (green shaded) at *t* = 1.98 s for pulsatile inlet flow of 4 L/min and frequency of 45 Hz. While the drug-laden air fraction is virtually identical to that of the non-pulsatile conditions (bottom and pie chart), the streamlines in pulsatile airflow have more complex structure after the nasal valve moving the air to different directions and streamlines of the non-pulsatile airflow moving straight after the nasal valve. The iso-volumes of variable C for values more than 0.99 from left and right views as well as close-up view of the left cavity are shown at the bottom. An iso-volume of variable C with values more than 0.5 is shown in the middle figure for a better visualization of values.

There are considerable similarities and some differences between pulsatile flow (Figure 7) and non-pulsatile flow profile (Figure 6) when comparing the passive scaler tracer contours. Air exchange in the olfactory region and the entire nasal cavity is virtually identical and almost complete (>97%) for both cases (bottom panel and pie chart in Figures 6, 7). One notable difference is the lower ventilation (lower C values) in the left cavity specifically at the nasal valve and vestibule region (Figure 7, bottom right) when pulsatile airflow is used. Moreover, the pattern of the streamlines after the nasal valve is more straight for the non-pulsatile condition. However, in the pulsatile airflow simulations, the streamlines are oriented into all directions due to the disturbance caused by pulsatile airflow pattern.

**FIGURE 8:**
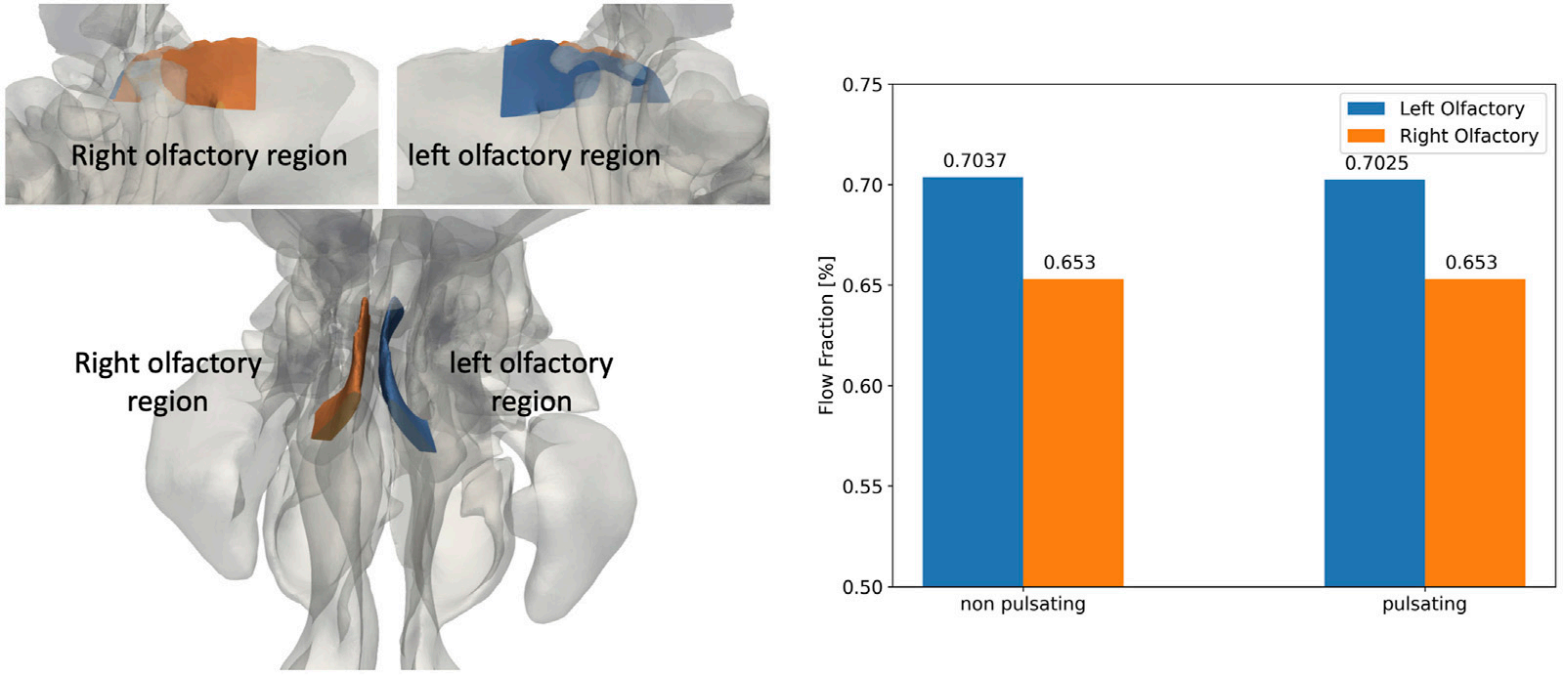
The geometry of the right (blue) and left (orange) olfactory regions (left figures) and the corresponding fraction of drug-laden airflow for non-pulsatile and pulsatile inlet airflow conditions (right plot) at the end of the inhalation phase (*t* = 1.98 s). The geometries of the olfactory regions are shown both from the side **(top left)** and the top **(bottom left).** The observed difference between flow fraction in the left and right olfactory region is due to corresponding differences in the left and right volume of the olfactory epithelium region, respectively.

A more detailed view on the olfactory region is presented in Figure 8. The fractional air flow in/through the left and right olfactory region at the end of the modeling period (*t* = 1.98 s) is 0.703 and 0.653% (of inhaled particle dose) independent of pulsation state, respectively, which is due to the slight differences in geometry and volume (left: 337 mm^3^; right: 331 mm^3^). Hence, from this perspective the conditions for relatively uniform left/right aerosol deposition in the olfactory epithelium region are favorable. This will be discussed in more detail in the next section.

### 3.4 Particles

Four million computational particles (parcels) with diameters of 1 µm are introduced for 0.5 s from the right nostril (at the velocity of the air at the nostril) and carried by non-pulsatile and pulsatile airflows (4 L/min) to the left nostril, which led to deposition of the particles in different regions of the cavity or to their exit through the left nostril. These bi-directional flow simulations are performed for a duration of 1.98 s to ensure that all particles have deposited or exited the nasal domain. Particle deposition patterns and DEs in the right and left nasal passages and olfactory regions are visualized and quantitatively evaluated at 1.98 s as presented below.

**FIGURE 9:**
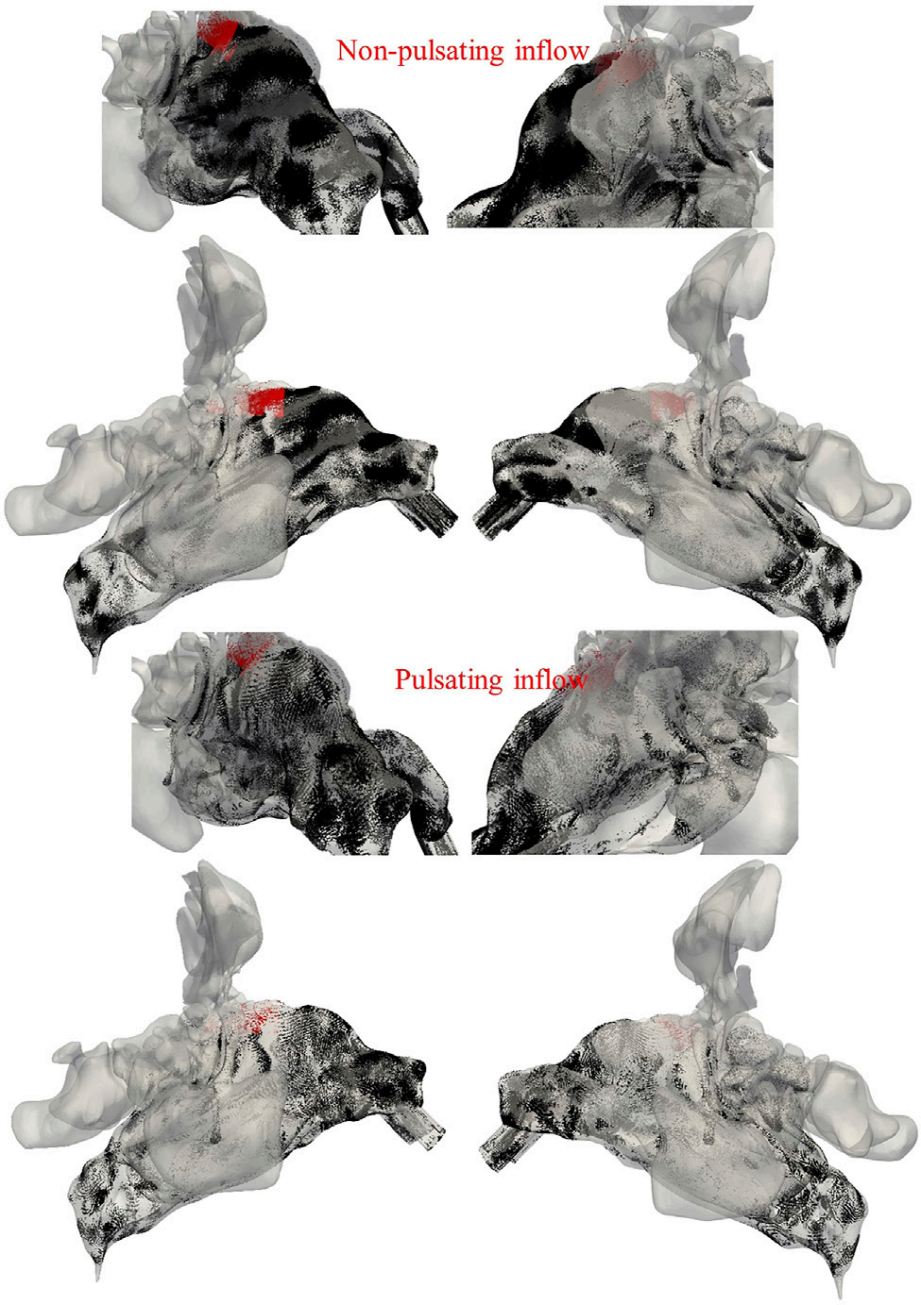
Deposition pattern of 1 µm particles (black spots) for non-pulsatile **(top)** and pulsatile inlet airflows **(bottom).** The particles deposited in the olfactory region are indicated by red color. Left and right panels refer to views onto the right and left side of the nasal cavity, respectively.

Figure 9 illustrates the spatial deposition pattern of 1 µm particles for either non-pulsatile (top) or pulsatile (bottom) inlet airflow in the nasal airway and olfactory region (highlighted). The main deposition occurs in the right nasal cavity and specifically in the regions with uneven surfaces which cause changes in the direction of the flow and consequently deposition of particles due to inertial impaction.

**FIGURE 10:**
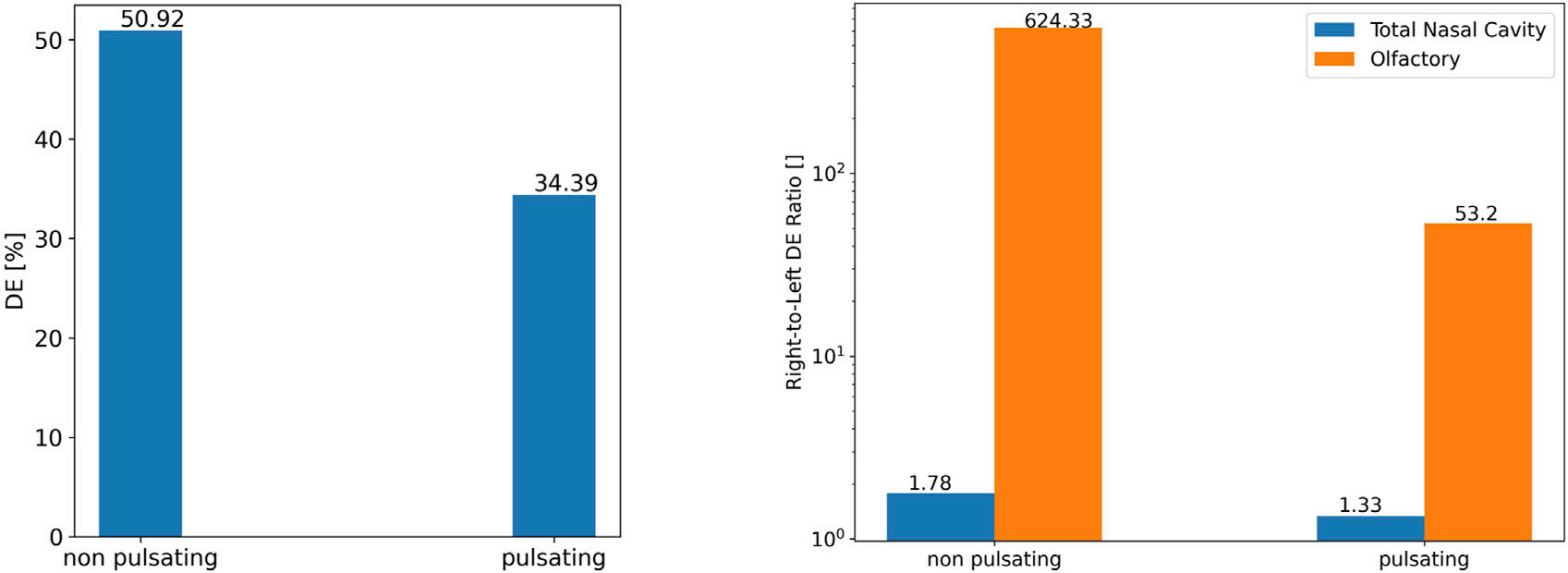
The role of pulsation on 1 µm particle deposition efficiency in the entire nasal cavity **(left)** and the right and left olfactory region (right: note: log scale).

Quantitative analysis of our model reveals that total (1 µm) particle deposition in the nose is 50.9 and 34.4% for aerosol delivery with non-pulsatile and pulsatile bi-directional 4 L/min air flow, respectively (Figure 10). This is significantly larger than the corresponding value for bilateral inhalation (1.25%) indicating that during normal inhalation there is almost no impaction in the nasal cavity, while for bi-directional flow the sudden change in flow direction in the nasopharynx region induces significant impaction of 1 µm particles. In bilateral aerosol delivery (injection from both nostrils) particles mainly escape the nasal passage from the nasopharynx to the pharynx. However, during bi-directional delivery, particles enter from one nostril and exit from the other one which leads to substantial enhancement of DE in the nasal cavity.

For further validation of particle deposition modeling beyond what has already been presented above, it is instructive to compare our CFD-derived total (1 µm) particle deposition in the nose (Figure 10) with empirical *in vitro* and other modeling data on bi-directional nasal aerosol deposition. Kleven et al. (2005) experimentally and numerically found total nasal DEs of 63.1 ± 18.4 and 45.4% for 3.5 µm particles bi-directionally inhaled with a constant flow rate of 6 L/min. Moreover, Xi et al. (2017) reported DE of 24 ± 6% for bi-directional nasal drug delivery with a constant inlet airflow rate of 6 L/min, particle size of 3.2 µm. In another *in vitro bilateral* aerosol delivery study with a PARI SINUS nebulizer (pulsatile flow, frequency 44.5 Hz), particle MMD of 3.79 ± 0.03 µm, and airflow rate of 6 L/min, DE for the adult nasal model was reported as 80.74 ± 10.17% (Hosseini and Golshahi, 2019). The large range of DEs in the literature are presumably an indication of the strong dependence of nasal aerosol deposition on nasal geometry and experimental conditions. The DE values of this study are in the range of the DEs reported in previous experimental and numerical studies on pulsatile and bi-directional flow. Flow pulsation not only reduces total aerosol deposition by a factor of 1.48 (from 50.9 to 34.4%), it also mitigates the non-uniformity of right-to-left cavity deposition from 1.78-fold to 1.33. Thus, pulsation reduces impaction-induced deposition in both cavities, but more pronounced in the right cavity. Similar but even more pronounced trends were observed for the olfactory region where pulsation reduced olfactory particle deposition from 0.48 to 0.12% of the inhaled aerosol (3.9-fold reduction). Even more striking, the right-to-left olfactory DE ratio was reduced from 624 for non-pulsatile to 53.2 and pulsatile airflow, respectively, i.e., pulsation reduces the right-left non-uniformity by more than one order of magnitude, but it is still severe. It is noteworthy that pulsation increased particles deposited in the left olfactory region by a factor of 4.4, albeit still on a very low level (<0.01%). The main reason for this extremely large difference between left and right olfactory deposition can be seen in Figures 4, 5. For the non-pulsatile flow, streamlines impinge on the top part of the cavity after the nasal valve, and the air passes with relatively high-speed through the right olfactory region.

The main reason for this extremely large difference between left and right olfactory deposition can be seen in Figures 4, 5. For the non-pulsatile flow, streamlines impinge on the top part of the cavity after the nasal valve, and the air passes with relatively high-speed through the right olfactory region. In contrast, due to the oscillating nature of the pulsatile flow, streamlines after the nasal valve are moderated and mixed resulting in lower air speed than that of the non-pulsating flow when passing through the right olfactory region. Only for the right nasal cavity, one can observe some streamlines going into the olfactory region, but none are going into the left olfactory region. Since reduced air flow towards the epithelium results in reduced impaction of particles, pulsation reduces particle deposition for all regions where particle deposition is mostly affected by convective transport (impaction) of particles.

For drug delivery purposes, one is typically not only interested in DE but also in the aerosol (drug) dose deposited onto the epithelium, since the local drug dose per area determines the therapeutic efficacy of a drug. Typically, only a fraction of the inhaled aerosol is due to drug, but for simplicity we will assume a 100% drug loading for the following discussion. Moreover, we assumed that an aerosol (drug) concentration of 75 g/m^3^ is inhaled at a flow rate of 4 L/min for 1.98 s yielding a total inhaled drug dose of 2.5 mg.

**FIGURE 11:**
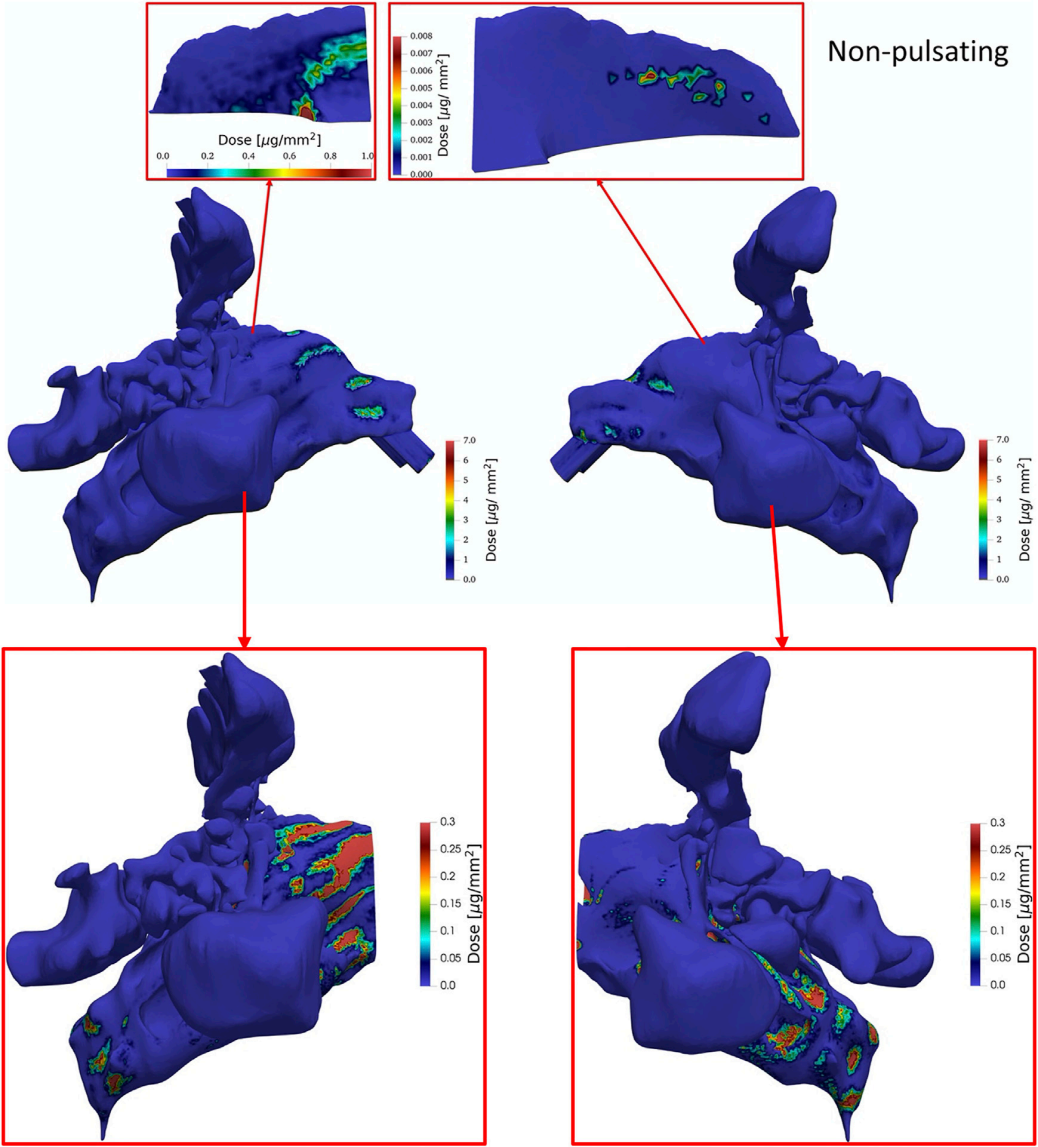
The contour plots of tissue-delivered particle dose (of 1 µm particles) presented as mass of particles per surface area tissue at *t* = 1.98 s (end of inhalation) in the entire nasal airway from right and left views **(middle)** as well as a reduced section of the nasal airway without the high dose section in the anterior part of the nasal cavity **(bottom)** and a close-up view of right and left olfactory regions **(top)** for non-pulsatile aerosol delivery with an aerosol concentration of 75 g/m^3^ (particle density assumed 1 kg/m^3^; water), constant flow rate of 4 L/min and a total inhaled dose of 2.5 mg.

The contour plots of Figure 11 illustrate the deposited drug dose represented as mass per area (epithelium) throughout the entire nasal cavity and specifically in the right and left olfactory regions when non-pulsating inlet airflow is used. The results show a patchy deposition pattern with the highest deposited dose regions in the anterior parts of the right nasal cavity specifically in the nasal valve and the upper parts after the valve. Less dose is observed in the nasal valve and vestibule of the left cavity compared to the right side. To gain a better understanding on the regional distribution of the deposited dose in the right and left olfactory regions, a close-up view of contour plots of dose is presented which shows a significantly higher dose in the right olfactory compared to the left olfactory epithelium region. The posterior nasal regions receive a much lower dose than the anterior region. Therefore, in separate figures these regions with corresponding dose values are shown. In the posterior region most of the deposition is observed where the flow direction changes or vortexes are formed, which is consistent with enhanced inertial impaction of the particles in regions where the air flow changes direction.

**FIGURE 12:**
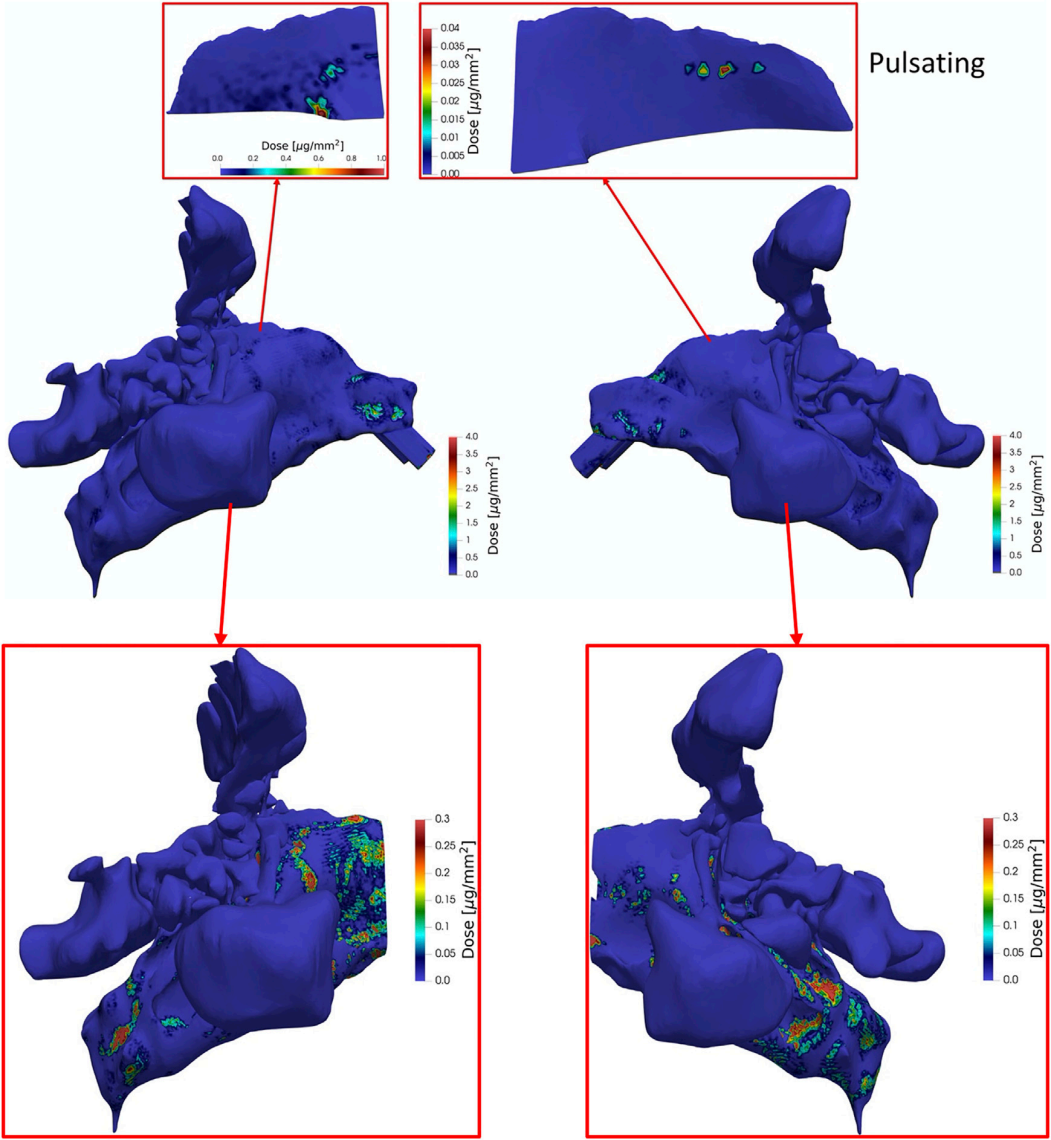
Same as Figure 11 except that pulsating aerosol delivery with pulsation flowrate of 4 L/min and frequency of 45 Hz at *t* = 1.98 s (end of inhalation) was applied.

Figure 12 is analogous to Figure 11, except that pulsatile aerosol delivery (4 L/min, 45 Hz) is presented. Pulsation of the airflow has almost completely avoided the deposition of particles along the direction of the nosepiece. Therefore, the dose in the regions that reside along the direction of the nosepiece is much lower compared to the condition that non-pulsatile (steady) aerosol delivery is used. In contrast to non-pulsatile aerosol delivery, there are no high dose regions in the upper parts after the nasal valve. However, similar to the non-pulsating aerosol delivery, the regions with highest doses are observed at the vestibule and nasal valve. Analogous to Figure 11 the dose in regions after the nasal valve are separately shown with their corresponding values. Pulsation of the airflow reduces the extreme accumulation of particles in the regions that stand as obstacle perpendicular to the flow direction.

**FIGURE 13:**
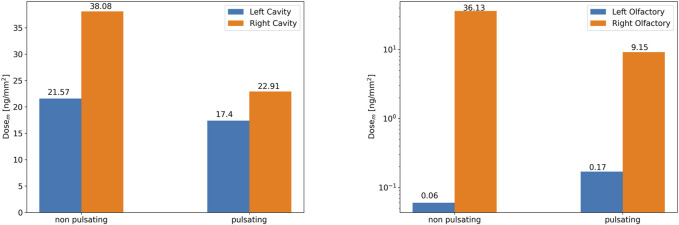
Comparison of average dose per area in the right and left nasal cavity **(left panel)** and the olfactory regions **(right panel)** for non-pulsatile and pulsatile aerosol delivery with average airflow rate of 4 L/min. The dose is presented as mass per area at *t* = 1.98 s (end of inhalation). The surface areas of the right **(left)** nasal cavities and olfactory regions are 21,393.1 mm^2^ (21,249.80 mm^2^) and 330 mm^2^ (337 mm^2^), respectively.

Analogous to Figure 10, average dose per area of epithelium in the nasal cavity is reduced due to pulsation. Figure 13 shows that there is almost no dose (<0.2 ng/mm^2^) deposited in the left olfactory region irrespective of pulsation conditions, while ca. 54–602-fold higher doses per area (9—36 ng/mm^2^) are delivered to the right olfactory region with pulsatile and constant flow conditions respectively. However, pulsatile airflow reduces the dose by a factor of ∼3.95 in the right olfactory region.

As seen from Figures 11, 12 and analogous to DE (Figure 10), pulsating flow reduces the right-left imbalance of deposited dose (per area) for both the entire cavity and the olfactory epithelium region. This is due to both reduced and enhanced aerosol deposition in the right and left half of the nose (both cavity and olfactory region), respectively. Albeit the DE in the right cavity (50.9 and 34.4%) is 68-fold and 162-fold higher than that in the right olfactory region for non-pulsating and pulsating flow, respectively (Figure 10), the average local dose (mass per area) in the right cavity is 1.05-fold and 2.5-fold higher than that in the right olfactory region for non-pulsating and pulsating conditions, respectively. This is mainly due to the ca. 64-fold lower area of the olfactory region as compared to nasal cavity. On the other hand, the local dose in the olfactory region is ca. 100-fold lower than that in the left cavity independent of pulsation conditions.

In summary, bidirectional inhalation of 1 µm aerosol provides relatively high nasal DE (>34.4%) and about the same average local (surface-specific) drug dose in the olfactory epithelium (ca. 36 ng/mm^2^) as observed in the nasal cavity for the right half of the nose (inhalation side). This is not the case for the left half of the nose, where the local aerosol dose in the olfactory regions is ca. 100-fold lower than in the nasal cavity. Hence, pulsatile flow did reduce the right-left imbalance in terms of DE, but not to levels where similar right-left local doses could be observed. Owing to the intricate nasal geometry the spatial aerosol deposition was highly non-uniform with very pronounced dose hotspots of up to 1,000 ng/mm^2^ even in the olfactory epithelium region (Figures 11, 12). Bidirectional aerosol delivery substantially enhanced the total nasal deposition efficiency for 1 µm particles carried by steady inhalation rate of 4 L/min compared to the conventional bilateral aerosol delivery method [50.9 versus ca. 1% (Shi et al., 2007; Shang et al., 2015)]. Similarly, bidirectional aerosol delivery enhanced the olfactory deposition efficiency compared to bilateral aerosol delivery with 1 µm particles [0.48 versus 0.1% (Si et al., 2013)].

The main reason for this extremely large difference between left and right olfactory deposition can be seen in Figures 4, 5. For the non-pulsating flow, streamlines impinge on the top part of the cavity after the nasal valve, and the air passes with relatively high-speed through the right olfactory region. In contrast, due to the oscillating nature of the pulsatile flow, streamlines after the nasal valve are moderated and mixed resulting in lower air speed than that of the non-pulsatile (constant) flow when passing through the right olfactory region. Only for the right nasal cavity, one can observe some streamlines going into the olfactory region, but none are going into the left olfactory region. Since reduced air flow towards the epithelium results in reduced impaction of particles, pulsation reduces particle deposition for all regions where particle deposition is mostly affected by convective transport (impaction) of particles. This is the case for all regions, except the left olfactory epithelium region where convection is virtually absent. Thus, we assume that in this region the oscillatory-flow-induced particle deposition, which has also been reported for non-ventilated paranasal sinuses (Möller et al., 2014), accounts for this finding. This almost complete lack of convective transport into the olfactory region (at least for the left side) can be interpreted as a “protective” mechanism of the olfactory nerve. This nerve is supposed to be sensitive to adsorbed smell-related molecules. Since these molecules reach the olfactory region quite effectively via diffusion (as indicated by large C values in this region, which is indicative of the diffusion of air molecules), the lack of convective transport protects the olfactory region from dust particles, which might deposit onto the olfactory tissue and impair the sensitivity of the olfactory nerve. This hypothesis would have to be substantiated by investigating nasal cavities from other individuals.

Hence, targeting of 1 µm particles to the olfactory tissue with bi-directional flow appears to be difficult. While pulsatile aerosol flow leads to a 3-fold enhanced deposition (DE) in the left olfactory epithelium region, there is a 3.9-fold DE reduction in the right one. The net effect is a more uniform aerosol deposition in the left and right olfactory region, but there is still a ca. 50-fold higher DE in the right olfactory region (inlet side). This suggests that the olfactory region is either not sufficiently conducive to acoustic resonance (which is possibly, since it is completely open on one side of the cavity, rather than fully enclosed with a small opening as required for a Helmholtz resonator) or its resonance frequency is too far away from 45 Hz. We consider the former more likely than the latter.

The effects of different parameters such as morphological differences in various individuals, different inlet flow rates, and particle sizes could play significant role in optimization of particle deposition in olfactory epithelium which should be optimized in future studies.

## 4 Conclusion

In the present study, a CT-based geometry of the nasal cavity and paranasal sinuses with open mouth (closed soft palate condition) was reconstructed and CFD simulations of bi-directional aerosol delivery w/o flow pulsation were performed. The modeling was validated with experimental data on generic bend geometries and actual measurements of realistic nasal aerosol deposition with normal (bilateral) flow (in casts).

Often computational aerosol deposition studies are only interpreted with respect to deposition efficiency (DE), but for therapeutic intervention the local deposited drug dose (mass per area) is more relevant. In this study, this revealed that, in spite of a relatively low DE (<1%) in the olfactory region, the local drug dose in the olfactory region was on a similar level (ca. 36 ng/mm^2^) as in the rest of the nasal cavity on the *inhalation* side owing to the ca. 64-fold smaller area covered by the olfactory region. As a caveat we mention that both in the olfactory and cavity region hot spot doses of up to ca. 1,000 ng/mm^2^ have been observed. While pulsative flow alleviated some of the right-left imbalance of aerosol deposition, a ca. 54-fold enhanced dose on the inhalation side (right) remained. On the other hand, pulsatile flow reduced the DE in both the nasal cavity and the olfactory region.

From this we conclude that pulsation does not provide a substantial benefit over bidirectional flow without pulsation. Bidirectional aerosol delivery substantially enhances DE both in olfactory region and total nasal airway compared to conventional bilateral aerosol delivery method.

These considerations also suggest two options for relatively efficient drug delivery (ca. 0.5% dose efficiency) to the olfactory region leveraging 1 µm aerosol and bi-directional constant flow conditions (no flow pulsation). (1) The olfactory region of the inhalation side of the nose can be targeted very effectively with a single bidirectional inhalation. (2) For efficient drug delivery to both sides of the olfactory region, two consecutive inhalations should be performed—once through each of the nostrils.

## Data Availability

The original contributions presented in the study are included in the article/supplementary files, further inquiries can be directed to the corresponding author.
